# A RESEARCHER–PRACTITIONER COLLABORATION ON MEASURING SUCCESS IN ELDER MISTREATMENT INTERVENTION

**DOI:** 10.1093/geroni/igad104.1571

**Published:** 2023-12-21

**Authors:** David Burnes, Patricia Kimball, Polly Cox, Kathryn Harnish, Carol Ayoob, M T Connolly, Geoff Rogers, Stuart Lewis

**Affiliations:** University of Toronto, Toronto, Ontario, Canada; Elder Abuse Institute of Maine, Brunswick, Maine, United States; Elder Abuse Institute of Maine, Brunswick, Maine, United States; Elder Abuse Institute of Maine, Brunswick, Maine, United States; Elder Abuse Institute of Maine, Brunswick, Maine, United States; University of Southern California, Los Angeles, California, United States; City University of New York, New York City, New York, United States; Dartmouth College, Lebanon, New Hampshire, United States

## Abstract

One of the major barriers to conducting elder mistreatment intervention research is a lack of understanding on how to measure outcomes of success. Within the context of the RISE project, this presentation will describe a highly collaborative process between researchers and RISE practitioners (supervisor and advocates) to develop a measurement instrument that meets validated research standards as well as the needs of practitioners who work directly on elder mistreatment cases and are responsible for administering the tool. Informed by ecological-systems, relational, and client-centered perspectives, RISE is a conceptually driven, evidence-based, community-based elder mistreatment response program that works with older adults at risk of or experiencing EM, as well as their alleged harmers, their relationship, and their surrounding social support systems. This presentation will describe the process of developing an appropriate measurement tool through weekly meetings over a 6-month period, from a starting point characterized by widely discrepant instrument expectations across the researcher-practitioner dyad toward an ending that achieved mutual agreement. This process required high levels of honesty, openness, patience, and humor among members. The final version of the measurement tool will be presented, which measures intervention outcome constructs of older adult social support, self-efficacy, life satisfaction, and perceived stress, as well as practitioner-client working alliance.

